# Quantitative Proteomic Analysis of Serum from Pregnant Women Carrying a Fetus with Conotruncal Heart Defect Using Isobaric Tags for Relative and Absolute Quantitation (iTRAQ) Labeling

**DOI:** 10.1371/journal.pone.0111645

**Published:** 2014-11-13

**Authors:** Ying Zhang, Yuan Kang, Qiongjie Zhou, Jizi Zhou, Huijun Wang, Hong Jin, Xiaohui Liu, Duan Ma, Xiaotian Li

**Affiliations:** 1 Obstetrics and Gynecology Hospital, Fudan University, Shanghai, China; 2 Children's Hospital, Fudan University, Shanghai, China; 3 Department of Chemistry, Fudan University, Shanghai, China; 4 Institute of Biomedicine, Fudan University, Shanghai, China; 5 Key Laboratory of Molecular Medicine, Ministry of Education, Department of Biochemistry and Molecular Biology, Institute of Biomedical Sciences, Shanghai Medical College, Fudan University, Shanghai, China; 6 Shanghai Key Laboratory of Female Reproductive Endocrine Related Diseases, Shanghai, China; I2MC INSERM UMR U1048, France

## Abstract

**Objective:**

To identify differentially expressed proteins from serum of pregnant women carrying a conotruncal heart defects (CTD) fetus, using proteomic analysis.

**Methods:**

The study was conducted using a nested case-control design. The 5473 maternal serum samples were collected at 14–18 weeks of gestation. The serum from 9 pregnant women carrying a CTD fetus, 10 with another CHD (ACHD) fetus, and 11 with a normal fetus were selected from the above samples, and analyzed by using isobaric tags for relative and absolute quantitation (iTRAQ) coupled with two-dimensional liquid chromatography-tandem mass spectrometry(2D LC-MS/MS). The differentially expressed proteins identified by iTRAQ were further validated with Western blot.

**Results:**

A total of 105 unique proteins present in the three groups were identified, and relative expression data were obtained for 92 of them with high confidence by employing the iTRAQ-based experiments. The downregulation of gelsolin in maternal serum of fetus with CTD was further verified by Western blot.

**Conclusions:**

The identification of differentially expressed protein gelsolin in the serum of the pregnant women carrying a CTD fetus by using proteomic technology may be able to serve as a foundation to further explore the biomarker for detection of CTD fetus from the maternal serum.

## Introduction

Congenital heart defects (CHDs) comprise the most common type of human birth defects, occurring in approximately one in 100 live births [1], [2], [3]. CHDs can be attributed to chromosomal and genetic abnormalities [4], [5], exposure to teratogens [6], maternal diabetes [7], maternal folate status, and folate-related genes [8]. Conotruncal heart defects (CTDs) account for 20–30% of CHDs [9], [10], [11], and affect the ventricular outflow tract and the arterial pole of the heart [12], [13], [14]. Only 20–25% of CTDs can be attributed to the above risk factors [15], most cases are nonsyndromic, with little known about their cause and risk. Currently there are no effective strategies for reducing the occurrence of CTDs, and no methods of early detection.

Serum protein screening is an important diagnostic tool, with a rich source, good sensitivity and simplicity. Proteins originating from the placenta, amniotic fluid or fetus circulation may cross the placenta barrier and exist in maternal serum. Currently, non-invasive procedures based on protein screening from maternal serum have been applied in the early screening of Down's syndrome [16], [17] by using the following serum markers: human chorionic gonadotropin (hCG), a-fetoprotein(AFP) [18], pregnancy-associated plasma protein (PAPP-A), unconjugated estriol (uE3) and inhibin-A [19]. An effective prenatal screening for CTDs is, however, lacking.

ITRAQ coupled with 2D LC-MS/MS appears a powerful technique in proteomics for identification of the protein quantitative changes caused by exposure or disease processes in cells, tissues or biological fluids [20], [21]. Tandem mass spectrometric analysis allows for the identification of multiple peptides per protein, providing increased confidence in both the identification and quantification of dysregulated protein. Recently, in many pathological pregnancies, proteomic technology has been used for the identification of differentially expressed proteins in amniotic fluid or maternal serum/plasma. These include screening for fetus' with abnormal karyotypes such as trisomy 21 [16], [22], [23], trisomy 18 [24], Turner syndrome [25], [26], Klinefelter syndrome (47, XXY karyotype) [27], intra-uterine growth restriction [28], [29], preeclampsia [29], [30], spontaneous preterm birth [31], [32] and intra-amniotic infection [33]. To the best of our knowledge, there are currently no reports on the application of proteomics to characterize differentially expressed proteins in maternal serum/plasma with CTD fetus.

In this study, we performed a relative quantitative comparison of differentially expressed proteins from the sera of women carrying a CTD fetus using iTRAQ combined with 2D LC-MS/MS, in order to explore potential screening markers of CTD fetus with sufficient sensitivity and specificity for clinical applications.

## Materials and Methods

### 1. Study population and design

This nested case-control study was carried out in the Obstetrics and Gynecology Hospital affiliated with Fudan University (Shanghai, China) between August 2009 and July 2010. 5437 pregnant women were enrolled, maternal peripheral venous blood samples were collected at 14–18 weeks of gestation, serum was isolated at 4°C and stored at −80°C for use. Prenatal ultrasound examinations were performed on all fetuses to screen for developmental abnormalities at 20–24 weeks of gestation; CTD, ACHD fetuses and normal controls were confirmed by prenatal and postnatal echocardiography or autopsy. Fetuses with chromosome abnormalities and multi-malformation, pregnant women with multiple pregnancies, pregnancy-related complications, abnormalities of cardiac structure and function or other comorbidities were excluded. Clinical information was collected for the established cases and controls. The controls were matched for (i) maternal age, (ii) gestational time at serum sample collection, (iii) gestational age at diagnosis by routine obstetric ultrasound, (iv) Number of pregnancies, and (v) parity (Table 1). Additionally, four heart tissues of CTD fetuses from the above cases and four heart tissues of normal controls from induced abortions because of unintended pregnancies were collected at 23–25 weeks of gestation and frozen at −80°C. The study was approved by the Research Ethics Board of Obstetrics and Gynecology Hospital affiliated with Fudan University, and participants gave signed written informed consent.

**Table 1:** Clinical characteristics of the pregnant women with a CTD, ACHD or normal fetus.

	CTD (n = 9)	ACHD (n = 10)	Control (n = 11)	*p*
Maternal age(years)	28.7±3.1	28.0±3.5	27. 0±3.2	NS
Gestational age for collection of serum(weeks)	16.9±1.1	16.1±1.5	16.4±1.1	NS
Gestational age at diagnosis by routine obstetric ultrasound(weeks)	22.5±1.0	22.3±1.3	22.5±1.3	NS
Number of pregnancies	2.0±1.3	2.2 ±1.	1.9±1.	NS
parity	1.1±0.4	1.2±0.	1.0±0.	NS

Statistical significant difference was not observed (one way-ANOVA) in three groups.

### 2. Depletion of high abundance proteins from maternal serum

Total protein content was determined in each serum sample, and samples from the same disease states were pooled with an equal protein amount to limit variability. Highly-abundant proteins were depleted by the Multiple Affinity Removal LC Column (4.6*50mm, Agilent Technologies, Inc, Palo Alto, CA) as per manufacturer's instructions. In each pooled sample, the high abundance proteins included albumin, immunoglobulin (Ig) A, IgG, IgM, transferrin, R1-acid glycoprotein, fibrinogen, α2-Macroglobulin, a1-antitrypsin, haptoglobin, apolipoprotein A-I, and apolipoprotein A-II. The depleted samples were then concentrated using centrifugal filter units, and the protein concentration was determined by the Bradford Protein Assay using a bovine serum albumin standard curve.

### 3. Protein digestion and iTRAQ labeling

Depleted protein samples were digested by using Acetone precipitation Ready Prep 2-D Cleanup Kit (Bio-Rad, Inc., CA). 100 µg of protein from each sample was dissolved in iTRAQ dissolution buffer according to the manufacturer's instructions (Applied Biosystems, Foster City, CA), reduced by 2 µl reducing reagent, incubated at 60°C for 1 h, alkylated in 1µl cysteine blocking reagent for 10 min at room-temperature and digested with trypsin at a ratio of 1∶20 overnight at 37°C. Digested samples were labeled with iTRAQ reagents following the recommended protocol. Isopropanaol was used to solubilize the iTRAQ isobaric tagging reagents. CTD group, ACHD group, and normal control were amino-labeled by iTRAQ reagents-118, 119, 121 respectively. The three samples were pooled, and the mixture of the trypsin-digested and iTRAQ-labeled samples was evaporated to dryness under vacuum and resuspended in acetonitrile (ACN) for the following MS analysis.

### 4. 2D LC–MS/MS analysis for protein identification and relative quantification

The labeled peptide mixture was fractionated by strong cation exchange (SCX) chromatography on an ultimate high-performance liquid chromatography (HPLC) system (Shimadzu, Kyoto, Japan) with a SCX column (2.1mm*100mm, 5µm, 200A, The Nest Group, Inc., MA). The mixed sample was suspended in buffer A (defined below) and loaded onto the column. Peptides were separated with a linear gradient of 0–80% buffer B in buffer A at a constant flow rate of 200 µl/min for 60 min. Buffer A consisted of 10 mM KH_2_PO_4_ and 25% ACN, pH 2.6 and Buffer B contained 10 mM KH_2_PO_4_, 25% ACN, 350mM KCl, pH 2.6. The chromatogram was monitored at 214 nm and 280 nm. 8 SCX fractions were collected along the gradient, and dried using a rotary vacuum concentrator (Christ RVC 2–25,Christ,Germany). The dried SCX peptides were dissolved in buffer C [5% ACN, 0.1% formic acid (FA)], prior to analysis on a QSTAR XL system (Applied Biosystems, Concord, ON, Canada). Peptides were loaded on a ZORBAX 300SB-C18 column (5 µm, 300 Å, 0.1×150 mm, Microm, USA) combined with the HPLC system at a flow rate of 0.3 µl/min for 90 min, and separated by C18 chromatography (75 µm ID, 100 Å) PepMap100 analytical column. The HPLC gradient ramped from 5% to 80% buffer D (95% ACN, 0.1% FA) in buffer C. The mass spectrometer data was acquired in information-dependent acquisition (IDA) mode with the m/z range set at 400–1800, and the four most intense peaks of were MS/MS scanned from m/z 100–2000. Data was acquired and collected with Analyst QS software (Version 1.1, service pack 8, AB/SCIEX).

### 5. Protein identification and quantitation

Both protein identification and quantification were performed by using the Paragon Algorithm in ProteinPilot software (version 3.0, Applied Biosystems). The settings were as follows: Sample Type, iTRAQ 8-plex (Peptide Labeled), Cys alkylation: methyl methane thiosulfonate (MMTS), digestion: trypsin, Instrument: QSTARESI, search effort: through ID, ID focus: biological modification, Database: non-redundant international protein index (IPI version 3.45 human). Protein ratio >1.40 or <0.71 was considered as potential differentially expressed proteins between diseased cases and controls for further investigation [34].

### 6. Western blotting

To validate the iTRAQ results, a total of 20 serum samples were screened by Western blot, including 9 CTDs and 11 normal controls, as previously described. The serum protein concentration was determined by a Bradford protein assay, with a bovine serum albumin (BSA) standard curve [35]. 30 µg of non-depleted serum proteins were loaded on a single lane and separated with 10% (or 12%) sodium dodecyl sulfate polyacrylamide gel electrophoresis (SDS-PAGE), with molecular weight standards run in parallel. The separated proteins were electrotransferred to nitrocellulose membranes at 4°C. The membranes were blocked for 2 h at room temperature with 5% non-fat milk in Tris-Buffered-Saline with Tween(TBST) and incubated with primary antibodies overnight at 4°C; the primary antibodies were rabbit polyclonal gelsolin (GSN) antibody (1∶75, Thermo, USA), mouse monoclonal ceruloplasmin (CERU) antibody (1∶1000, Abcam, Hong Kong), mouse monoclonal Attractin(ATRN) antibody (1∶1000, Abcam, Hong Kong), goat monoclonal Alpha-2-macroglobulin(A2M) antibody (1∶5000, Thermo, USA), goat polyclonal Pregnancy zone protein(PZP) antibody(1∶150 SANTA CRUZ, USA), respectively. After washing with TBST in triplicate, the blots were incubated in HRP-conjugated secondary antibody for 1 h at room temperature. Immunoreactive proteins were detected with SuperSigal West FemtoMaximun Sensitivity Substrate (Thermo, USA), and then exposed to X-ray film. The densitometry of the bands was estimated by Quantity One.

The GSN expression in the four heart tissues of CTD fetus and four normal controls were also analyzed using Western blot. The tissues were directly lysed with a fixed volume of cell lysis buffer (50mM Tris-HCl, pH 7.4, 150mM NaCl, 1% Triton-100), followed by vortexing for 10 minutes. Protein inhibitor was added during cell lysis. 20 µg of heart proteins were loaded on a single lane and separated with 10% SDS-PAGE and electrotransferred to nitrocellulose membranes. Blots were incubated with primary GSN antibody (1∶75, Thermo, USA), followed by incubation with secondary antibody. GAPDH was used as a loading control. Triplicate blots were carried out for each tissue sample to ensure robustness of the data generated.

### 7. Immunohistochemistry

Fetal heart tissues were examined by immunohistochemistry as described previously. Sections of heart tissue were prepared from paraffin embedded fresh tissues, blocked for 1 h at room temperature with 1% bovine serum albumin in PBS and incubated with primary rabbit polyclonal GSN antibody (1∶100, Thermo) overnight at 4°C and goat anti-rabbit HRP-conjugated secondary antibody for 1 h at room temperature. Pictures were analyzed under a phase microscope at a 400× magnification.

### 8. Statistical analysis

Data were expressed as mean ± SD. Mann–Whitney U-tests or one-way ANOVA was used to determine the significant difference of variables between two groups or three groups. Statistical analyses were performed using the SPSS19.0 software (SPSS Inc. Chicago, IL, USA). *p*<0.05 was considered statistically significant.

## Results

### 1. Derivation of the study population

**Figure 1:**
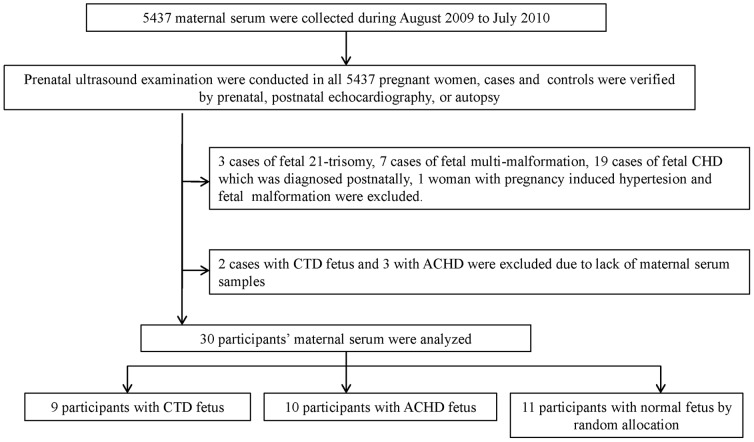
Flow diagram describing the selection of the analytical data set used for iTRAQ analysis.

Figure 1 displays the division of the analytical data set. 12 fetuses were diagnosed with isolated CTD, 15 fetuses with isolated ACHD and 7 with multi-malformation by ultrasound examination at 20-24 weeks of gestation. Termination of pregnancy was chosen in 7 of CTD cases after diagnosis, and CTD was verified as an isolated defect by autopsy. Other CTDs, ACHDs and normal controls were confirmed by prenatal and postnatal echocardiography. 3 cases of fetal 21-trisomy, 7 cases of fetal multi-malformation, 19 cases of fetal CHD which was diagnosed at birth, and 1 case combined with pregnancy–induced hypertension and fetal malformation were excluded. After exclusion of these women and those whose serum samples were lost, 9 participants with CTD fetus, 10 participants with ACHD fetus, 11 participants with normal fetus were recruited. Table 2 shows the types of prenatally diagnosed CTDs and ACHDs from the collected samples.

**Table 2:** Types of congenital heart defects included in this study.

Types of congenital heart defects	Number
CTD	
Tetralogy of Fallot (TOF)	4
Truncus arteriosus TA	1
Transposition of the great arteries (TGA)	2
Double-outlet right ventricle (DORV)	1
Pulmonary atresia (PA)	1
ACHD	
Ventricular septal defect (VSD)	6
Atrial septal defect (ASD)	1
Complete atrioventricular septal defect (CAVSD)	2
Complete endocardial cushion defect with uniatrium	1

### 2. The identification of differentially expressed maternal serum proteins in women carrying a CTD fetus and ACHD fetus by iTRAQ and 2D LC/MS/MS analysis

**Figure 2:**
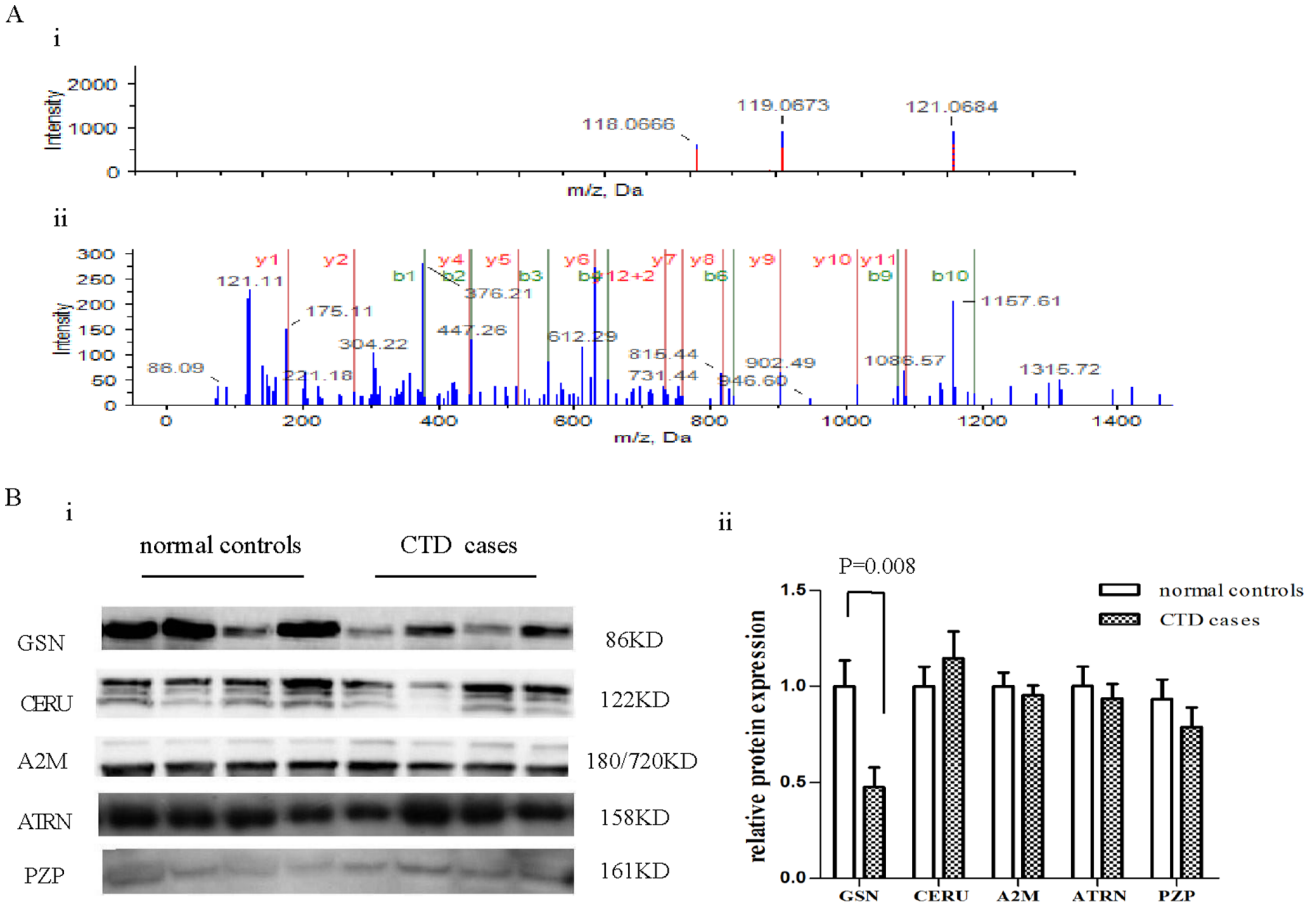
Identification of the protein gelsolin as being differentially expressed in maternal sera of pregnant women carrying a CTD fetus. (A) Identification of the protein gelsolin in maternal serum by iTRAQ and 2D LC-MS/MS. (i) The peptide quantitation information of gelsolin was derived from the intensities of three iTRAQ reporter ions. Reporter ions 118,119 were used to label sera from pregnant women carrying CTD and ACHD fetuses, respectively. Reporter ion 121 was used to label normal controls. (ii)The representative MS/MS spectrum of a peptide from gelsolin. (B). The Western blot validated the relative expression of GSN, A2M, CERU, ATRN, PZP in maternal serum samples(i), and confirmed relative decreased level of maternal serum gelsolin in CTD group compared with normal controls (ii). n = 9 in CTD group, n = 11 in normal control.*p = 0.008.

A total of 105 unique proteins present in the three groups were identified, and relative expression data were obtained for 92 of them with high confidence by employing the iTRAQ combined with 2D LC/MS/MS technologies. The information of identification and quantification is provided in Table S1. The detailed information for one differentially expressed protein-gelsolin (GSN) is shown in Figure 2A.

The criteria of a differential abundance of 1.40 and 0.71 fold changes were applied to the 92 identified proteins, and a total of 13 proteins showed significant changes between the two CHD groups and control group. Compared to the normal controls, 11 proteins in CTD group were dysregulated, with 3 upregulated and 8 downregulated proteins. Meanwhile, 7 proteins in the ACHD group were dysregulated, with 2 elevated and 5 depleted proteins. Of the dysregulated proteins, 5 proteins were shared between the CTD and ACHD groups, and 6 proteins were differentially expressed only in the CTD groups, including Ceruloplasmin(CERU), Complement factor B(CFAB), Pregnancy zone protein(PZP), Gelsolin (GSN), Alpha-2-macroglobulin (A2M), Attractin (ATRN). The full list of differentially expressed proteins can be found in Table 3.

**Table 3:** Maternal serum proteins showed differently expression in CTD group and ACHD group compared to normal control (CON).

Accession number	Gene	Protein	Function	CTD/CON		ACHD/CON	
				Ratio	*p*	Ratio	*p*
P0C0L5	CO4B	Complement C4-B	Complement activity	0.3565	0.0005	0.4875	0.0001
P00450	CERU	Ceruloplasmin	oxidoreductase activity	0.6546	0.0371	0.8630	0.6704
P02751	FINC	Fibronectin	intracellular protein transport	0.9204	0.9958	0.5754	0.0078
P00751	CFAB	Complement factor B	Unknown	0.6792	0.0010	0.7178	0.0155
P19827	ITIH1	Inter-alpha-trypsin inhibitor heavy chain H1	serine protease inhibitor	0.6546	0.0582	0.6427	0.0265
Q14624	ITIH4	Inter-alpha-trypsin inhibitor heavy chain H4	serine protease inhibitor	0.5297	0.0310	0.6026	0.0143
P20742	PZP	Pregnancy zone protein	cytokine activity	0.2884	0.0021	1.3062	0.1249
P06727	APOA4	Apolipoprotein A-IV	lipid transporter activity	0.4529	0.0195	0.6792	0.0130
P06396	GSN	Gelsolin	structural constituent of cytoskeleton	0.4656	0.0009	0.8630	0.3166
P01023	A2MG	Alpha-2-macroglobulin	cytokine activity	0.6138	0.0300	0.7586	0.5209
P43652	AFAM	Afamin	transport	2.7542	0.0378	2.6546	0.0431
P02787	TRFE	Serotransferrin	serine-type peptidase activity	3.9084	0.0228	11.1686	0.0008
O75882	ATRN	Attractin	oxidoreductase activity	1.4723	0.0493	0.9727	0.9742

### 3. Western blot validation of the protein expression in maternal serum

Except of the protein CFAB whose antibody cannot be available, the other five proteins, including CERU, GSN, A2M, ATRN, PZP were verified using Western blot (Figure S1). Figure 2B displays a summary of the protein expression from the maternal sera of 9 women carrying CTD fetus in comparison to that of 11 women carrying normal fetus, detected by Western blot analysis. GSN expression was downregulated in both iTRAQ and Western blot, in the maternal sera of women carrying a CTD fetus compared to those of the normal controls (*p* = 0.008). A2M, CERU, ATRN, PZP were not significantly differentially expressed (*p*>0.05).

### 4. Western blot and IHC validation of the gelsolin protein expression in fetal heart tissue

**Figure 3:**
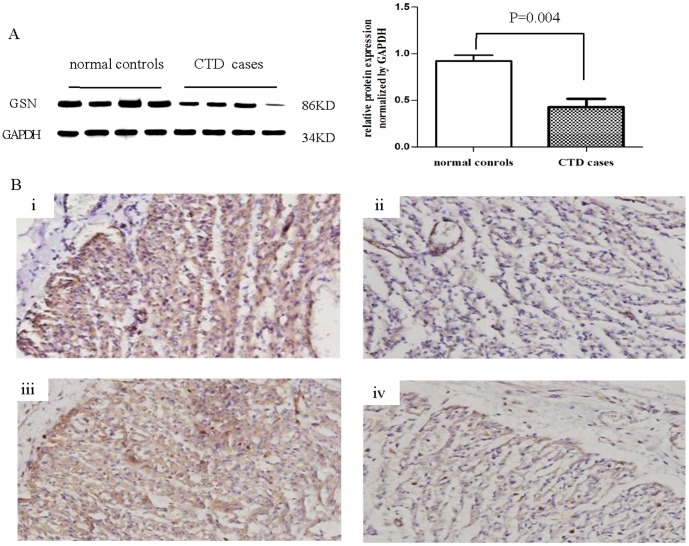
Western blot and IHC analyses confirmed the relative expression of GSN in fetal heart tissues with CTD. (A) Western blot analysis examined the relative expression of GSN, and confirmed that the GSN protein was downregulated in heart tissues of CTD fetuses compared with normal controls. n = 4, respectively. *p = 0.004. (B) IHC demonstrated the dark brown immunostaining was weaker in the CTD heart tissues than the normal controls (i, normal control, 23 gestational weeks, ii, CTD 23 gestational weeks; iii normal control, 25 gestational weeks, iv, CTD 25 gestational weeks). n = 4, respectively. Magnification was set at 400×.

Having validated that GSN protein was downregulated in maternal serum with a CTD fetus, we also examined the expression of GSN in 4 gestation week-paired CTD fetal hearts (23–25 gestational weeks), and 4 normal controls by Western blot and IHC. Figure 3A demonstrates that the GSN expression in heart tissues of fetuses with CTD in comparison with normal tissue, detected by Western blot. The expression of GSN was downregulated in CTD fetuses in comparison with normal controls. Figure 3B shows the expression level and localization of GSN in heart tissues of the representative cases by IHC. GSN protein is expressed in the cytoplasm of myocardiocytes. The dark brown immunostaining is weaker in the CTD heart tissues than the normal controls, further verifying the downregulation of GSN in CTD fetus' heart (i, normal control, 23 gestational weeks, ii, CTD 23 gestational weeks; iii normal control, 25 gestational weeks, iv, CTD 25 gestational weeks).

## Discussion

In this nested-control study, we identified the protein gelsolin as being differentially expressed in the sera of pregnant women carrying a CTD fetus at 14–18 gestational weeks. In comparison with sera from women carrying a normal fetus, gelsolin was significantly downregulated in the CTD group, as validated by proteomic analysis and Western blot. Moreover, decreased level of gelsolin in CTD fetal heart tissue was confirmed. It provided a valuable clue to search for the potential pathogenesis of CTD and develop a predictor of CTD.

As the prognosis of CTD infants is much poorer than that of other CHD(such as ventricular septal defect, etc) infants, we specially focused on the differentially expressed proteins of CTD in this study. Our study used pooled samples after depletion of the highly abundant proteins for iTRAQ labeling, which worked effectively for detecting low abundance proteins and avoiding clogging to the LC columns by the unprocessed serum [31]. However, pooled sample beforehand could mask the inter-sample variability, so we validated each sample by Western blot. Abnormal cardiac development usually occurs before 8 weeks of gestation, which is much earlier than ultrasound detection of CTD at around 24 gestational weeks. The environmental pathogenic factors for early cardiac development in maternal serum may appear earlier than abnormities can be detected by ultrasound, therefore, we collected the maternal serum at 14–18 gestational weeks, as near to the onset of CTD as possible. Currently, some markers (such as brain natriuretic peptide (BNP) and N-terminal pro-brain natriuretic peptide (NT-proBNP)) have been proven to be useful for the diagnosis of complex and significant congenital heart disease in neonates, children and adults [36], [37], [38], however, biomarkers for detection complex congenital heart disease in the fetus have not been reported.

Gelsolin is a Ca^2+^-dependent actin filament severing and capping protein which is involved in multiple important biological and clinical functions. Low expression of gelsolin has been observed in many cancers, including ovarian [39], breast [40], colon [41] and prostate [42], cervical [43]. Plasma gelsolin level also decreases in many critically ill patients such as burn. [44], sepsis [45], traumatic brain injury [46], [47]. The reduced level in plasma gelsolin is positively associated with the severity of these diseases, which can be used to predict the prognosis of diseases. The expression change of gelsolin in heart tissues is closely related to cardiac injury. The expression of gelsolin is upregulated in failing human hearts [48], pressure overload myocardium [49], and the gelsolin overexpression could induce cardiac hypertrophy [50]. The downregulation of gelsolin is a prosurvival factor for inhibition of heart failure progression after myocardial infarct. Gsn^-/-^ mice had a lower mortality, reduced hypertrophy, less interstitial fibrosis and improved cardiac function compared with Gsn^+/+^ mice after they were subjected left anterior descending coronary artery ligation. Gelsolin exists in midtrimester amniotic fluid of normal pregnancy and inhibits the proinflammatory immune response [51]; and serum gelsolin concentration was decreased with gestational age during normal pregnancy [52]. The expression of gelsolin was found to be downregulated in amniotic fluid supernatants from Klinefelter syndrome fetuses [27], and in maternal plasma of fetuses with Down Syndrome [23], preeclampsia and intra-uterine growth restriction [29]. However, the validations of proteomic results have not yet been reported for these pathological pregnancies. In this study, the decreased gelsolin level in the sera of women carrying a CTD fetus was verified by Western blot for the first time. But, the exact mechanism for this result is still unclear, which is probably of maternal origin itself, or may be caused by reduced secretion from placental and amniotic fluid. The plasma levels of gelsolin in tetralogy newborns were significantly lower than those in normal controls [53], so it is also plausible that the decreased gelsolin in the CTD fetus' circulation is a potential source of that in maternal serum. Interestingly, we found that gelsolin was also downregulated in the heart tissue of CTD fetuses by Western blot and IHC. Gelsolin knockout mice (Gsn-/-) are not lethal, and gelsolin is expressed in the myocardium of the atria and left ventricle from E10.5 days which is a critical time point in heart chamber morphogenesis and early septal development instead of outflow tract [54], thus, decreased expressed of gelsolin may not directly be a risk factor of CTDs. Further studies are needed to explore the reason of the decreased gelsolin level in the sera of women carrying a CTD fetus and the effect of low gelsolin level in maternal serum on development of embryo.

Currently, ultrasound scan and echocardiography are the major tools for CTD fetal prenatal diagnosis [11], [55], however, the first ultrasound examination is usually beyond 20 gestational weeks. The accurate diagnosis of such conditions requires special facilities, highly qualified operators, and the correct prenatal assessment of the ventricular septum or the distal aortic arch is difficult [55], [56]. Furthermore, many hospitals in China can not develop the routine fetal ultrasound scan and echocardiography. There is therefore an increasing demand for the development of a new method for earlier screening of fetal CTDs. Although the reason for decreased expression of gelsolin in maternal serum with CTD fetus is not still understood, the detection of gelsolin from maternal serum is non-invasive, requires small amounts of sample, and we believe that this study may provide an independent, low cost and complementary strategy for screening of fetal CTD. However, the confirmation of gelsolin protein as a potential screening biomarker requires validation of this protein level in all kinds of pregnancies, with a larger sample size. Furthermore, it is necessary to set a large–scale, systematic, prospective experiments for elucidating the sensitivity and specificity for population screening.

In summary, we successfully detected decreased levels of the protein gelsolin in the maternal sera of pregnant women carrying a CTD fetus in comparison with normal controls using iTRAQ labeling combined with 2D LC–MS/MS. The downregulation of gelsolin associated with CTD fetuses was confirmed by Western blot, both in sera and fetal heart tissues with CTD. Future studies should assess the quality of gelsolin as a maternal serum biomarker of fetuses with CTD.

## Supporting Information

Figure S1 The Western blot confirmed relative decreased level of maternal serum gelsolin in CTD group compared with normal controls. n = 9 in CTD group, n = 11 in normal control. *p = 0.008.
(TIF)

Table S1 The information of identification for 105 proteins and relative quantification for 92 proteins present in the three groups obtained by iTRAQ combined with 2D LC/MS/MS. 118, 119, 121 were used to represent CTD group, ACHD group, and normal control, respectively.
(XLSX)
