# Supplementary figures and images for: Quantitative Proteomic Analysis of Serum from Pregnant Women Carrying a Fetus with Conotruncal Heart Defect Using Isobaric Tags for Relative and Absolute Quantitation (iTRAQ) Labeling
Source: PLoS One. 2014 Nov 13;9(11):e111645. doi: 10.1371/journal.pone.0111645 (PMC4230941; doi:10.1371/journal.pone.0111645)

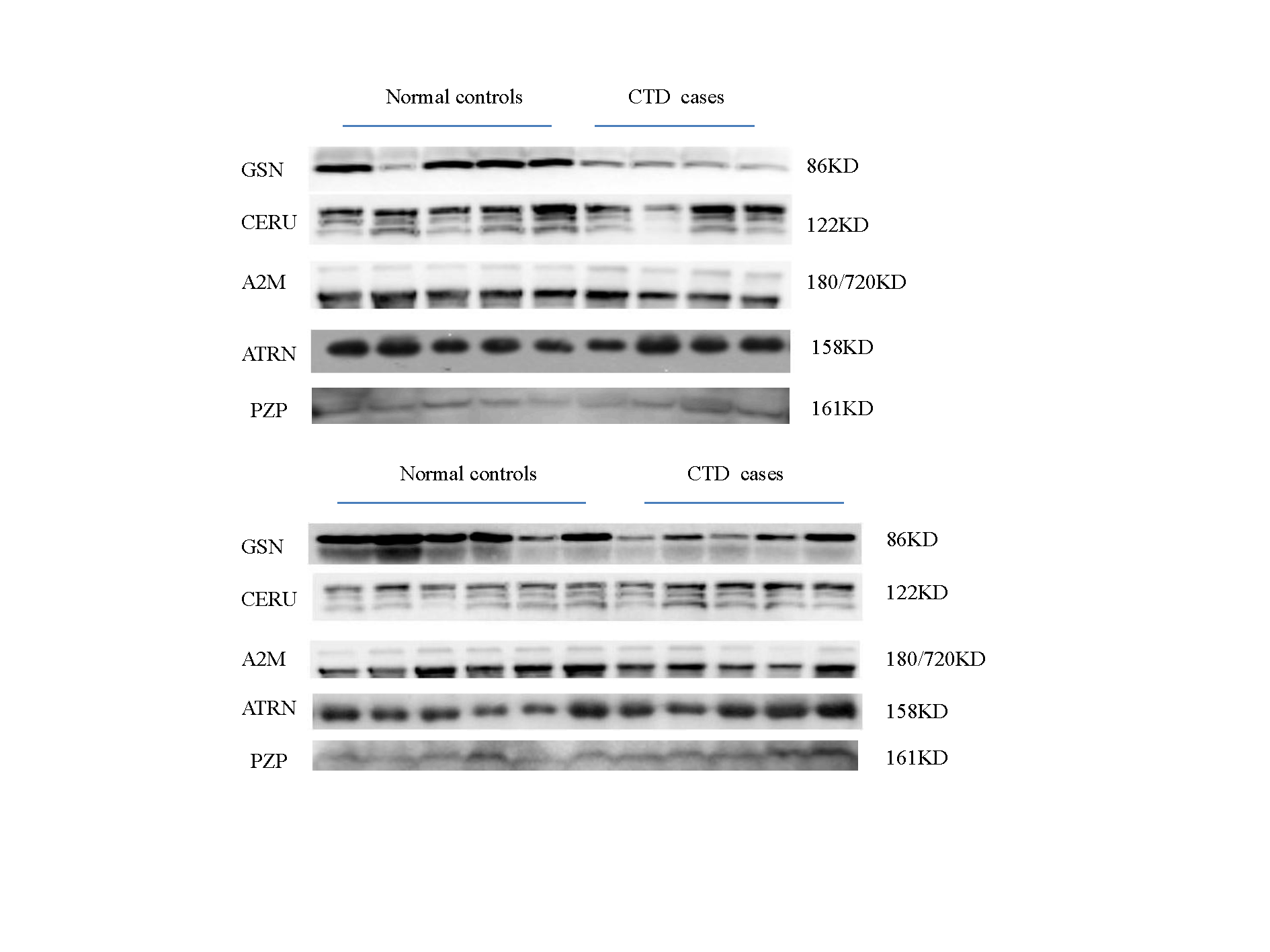

Supplement: Figure S1 — The Western blot confirmed relative decreased level of maternal serum gelsolin in CTD group compared with normal controls. n = 9 in CTD group, n = 11 in normal control. *p = 0.008. (TIF) [file pone.0111645.s001.tif]
